# Strategies for surface coatings of implantable cardiac medical devices

**DOI:** 10.3389/fbioe.2023.1173260

**Published:** 2023-05-09

**Authors:** David Coronel-Meneses, Calef Sánchez-Trasviña, Imma Ratera, Karla Mayolo-Deloisa

**Affiliations:** ^1^ Tecnologico de Monterrey, The Institute for Obesity Research, Monterrey, Mexico; ^2^ Tecnologico de Monterrey, Escuela de Ingeniería y Ciencias, Centro de Biotecnología-FEMSA, Monterrey, Mexico; ^3^ Institute of Materials Science of Barcelona (ICMAB-CSIC), Campus UAB, Bellaterra, Spain; ^4^ Centro de Investigación Biomédica en Red de Bioingeniería, Instituto de Salud Carlos III Bellaterra, Spain

**Keywords:** cardiac medical devices, surface coatings, biomaterials, biocompatibility, infections

## Abstract

Cardiac medical devices (CMDs) are required when the patient’s cardiac capacity or activity is compromised. To guarantee its correct functionality, the building materials in the development of CMDs must focus on several fundamental properties such as strength, stiffness, rigidity, corrosion resistance, etc. The challenge is more significant because CMDs are generally built with at least one metallic and one polymeric part. However, not only the properties of the materials need to be taken into consideration. The biocompatibility of the materials represents one of the major causes of the success of CMDs in the short and long term. Otherwise, the material will lead to several problems of hemocompatibility (e.g., protein adsorption, platelet aggregation, thrombus formation, bacterial infection, and finally, the rejection of the CMDs). To enhance the hemocompatibility of selected materials, surface modification represents a suitable solution. The surface modification involves the attachment of chemical compounds or bioactive compounds to the surface of the material. These coatings interact with the blood and avoid hemocompatibility and infection issues. This work reviews two main topics: 1) the materials employed in developing CMDs and their key characteristics, and 2) the surface modifications reported in the literature, clinical trials, and those that have reached the market. With the aim of providing to the research community, considerations regarding the choice of materials for CMDs, together with the advantages and disadvantages of the surface modifications and the limitations of the studies performed.

## 1 Introduction

Worldwide Cardiovascular diseases (CVDs) are cataloged as one of the main causes of morbidity and mortality. Each year more than 17.9 million people die from heart problems, which in turn represents 32% of all deaths worldwide, and this number is expected to increase exponentially by the year 2030 (De Hert et al., 2018; Timmis et al., 2022). Prevention and correct diagnosis are critical to reducing the number of annual deaths from heart problems. CVDs events could potentially be prevented if adults attain high cardiovascular health by addressing behavioral risk factors such as tobacco use, obesity, harmful use of alcohol, and low hemoglobin levels (van Iterson et al., 2018; Papanastasiou and Giannakoulas, 2021). Subsequently, the implementation of cardiac medical devices (CMDs) has become highly relevant to increasing patients’ life expectancy (American Heart Association, 2022).

CMDs become necessary when a patient has compromised cardiac capacity and/or activity related to the failure of one of the four heart valves (Karimov et al., 2020). The use of CMDs is widening abruptly. From 1950, when the first pacemaker (PM) was inserted; to 2022, more than 4.2 million CMDs have been implanted in US (American Heart Association, 2022). Nowadays, there are different CMDs to treat multiple cardiac complications, within which we can highlight: 1) valvular heart disease: surgical bioprosthetic and mechanical heart valves; 2) cardiac arrhythmias: cardiac pacemakers, implantable cardioverter-defibrillators (ICDs); 3) congestive heart failure: percutaneous mechanical circulatory devices and durable ventricular assist devices (VADs) and total artificial hearts (TAH); and 4) atrial septal defects and atrial fibrillation: closure devices and left atrial appendage occlusion devices (Karimov et al., 2020). All these CMDs are made of different materials; however, the selection of materials to build up CMDs is not trivial.

During the development of CMDs, multiple considerations must be made to select the proper materials: biocompatibility/hemocompatibility, mechanical properties, toxicity, infection, surface properties, degradability, and cost (Alipour et al., 2022). Among the main considerations in the formulation of CMDs, biocompatibility is a fundamental aspect that must be acknowledged in CMDs for the short-term and long-term (Ufukerbulut and Lazoglu, 2011). In this context, titanium and nitinol (NiTi) are the main materials used in the formulation of implantable medical devices (e.g., orthopedics, vascular stents, orthodontics, and essential parts of CMDs) (Pelton et al., 2000; Stoeckel, 2000). Furthermore, in recent years nitinol has been the most widely used material for formulating of multiple cardiac devices (Ufukerbulut and Lazoglu, 2011; Ahmed et al., 2020). NiTi has numerous benefits, such as extremely low cytotoxicity, excellent biocompatibility, memory effect, super elasticity, and high damping (Pelton et al., 2004; Alipour et al., 2022; Nagaraja et al., 2022). Despite increasing patients’ life expectancy and quality of life, infections and biocompatibility issues in CMDs have emerged in recent years (Timmis et al., 2022).

Several factors have been reported to be associated with a greater risk of CMDs failure: immunosuppression (e.g., renal dysfunction and corticosteroid use), oral anticoagulation use (e.g., type of anticoagulant, wrong dose, or overdose), coexisting patient illnesses, and bloodstream infection, particularly *Staphylococcus aureus* coagulase-negative (Freixa et al., 2003; Bongiorni et al., 2012; Blomstrom-Lundqvist and Ostrowska, 2021; Perrin and Deharo, 2021).

Infections in CMDs can be classified as local infections or systemic infections. Local infections are associated with a specific device manipulation (e.g., implantation, revision, or replacement). On the other hand, systemic infections are associated with the infection of the intravascular parts of the device (e.g., leads, or wires, that connect the device to the heart) (Blomstrom-Lundqvist and Ostrowska, 2021). It has been proclaimed that 55% of local infections occur during the first months after implantation (American Heart Association, 2022). Therefore, it is necessary to develop coatings on the surface of the material to prevent infections and enhance the biocompatibility of the CMDs.

Surface modification, with the immobilization of bioactive molecules, is the most commonly used and effective method to strengthen the biocompatibility and avoid infections (Nie et al., 2016; Yang et al., 2018; Kazemzadeh-Narbat et al., 2021; Sánchez-Bodón et al., 2022). Among the surface modifications used for CMDs, the following are highlighted: 1) modification of passive surface, 2) bioactive surface, 3) anticoagulant surface, 4) functionalization with platelet inhibitors or 5) fibrinolytic agents, and 6) surfaces that promote endothelialization. All these surface modifications are intended to enhance biocompatibility by using bioactive compounds attached to the surface or realizing bioactive compounds, allowing the CMDs to increase their biocompatibility and avoid rejection (Kazemzadeh-Narbat et al., 2021).

The current similar literature reviews content related to surface modifications in CMDs, which are analyzed and compared to the materials employed in developing CMDs. Their main objective is solving biocompatibility/hemocompatibility or infection issues, but not addressing both by surface modifications (Sin et al., 2012; Stanisławska, 2014; Festas et al., 2020; Zhao and Feng, 2020; Ahadi et al., 2023). The following review outlines not only the materials employed in the formulation of CMDs but how to avoid biocompatibility/hemocompatibility issues and infections using surface modifications. To achieve this, first, the main cardiac problems that may lead to a CMD need are detailed. In addition, a critical comparison between the multiple materials used in the development of CMDs is presented. Following this, problems related with the use of CMDs are summarized, highlighting biocompatibility and hemocompatibility. The fourth section, the central part of this review, presents a critical analysis of the multiple surface modifications in CMDs reported in the literature. Some surface modifications described are still in clinical trials, but others have already been approved for use in clinics. Finally, a choice-making guide is outlined for selecting a suitable surface modification of CMDs.

## 2 Cardiac pathologies and their medical devices

The heart rate of healthy adults ranges between 60 and 100 beats per minute in a resting state (Camm et al., 2018). To carry out this task, the human heart must complete an entire cycle, which comprises two main phases: 1) diastole phase (Burkhoff et al., 2005); and 2) systole phase (Powers and McCulloch, 2022). The human heart, a set of muscles working together, tends to suffer degeneration as time progresses and workload increases (Chien and Karsenty, 2005; Groenewegen et al., 2020; Karimov et al., 2020). However, it is noteworthy that these cardiac anomalies (coronary artery diseases, valve problems, cardiac arrhythmias, congestive heart failure, atrial septal defects and atrial fibrillation) can appear before an advanced age (around 60 years old) due to multiple factors, such as genetic predisposition (Roselli et al., 2020), congenital abnormalities (Hoffman and Kaplan, 2002; McElhinney et al., 2003) or an inappropriate lifestyle (Dominic et al., 2022) arising the need to the implementation of CMDs.

Coronary arteries carry blood to the heart and can be blocked by atherosclerotic plaque. Atherosclerotic plaque, principally made of deposits of cholesterol, causes the interior of the coronary artery to stretch, which can either wholly or partially obstruct blood flow (Borhani et al., 2018; Ahadi et al., 2023). One way to solve coronary artery diseases is by introducing stents. Stents are made of metal alloys (see Table 1) (Borhani et al., 2018). The selection of these materials is based on the following characteristics: they need to be resorbable, flexible, non-erodible, non-cytotoxic, and biocompatible (Borhani et al., 2018). Moreover, intravascular injuries result in in-stent restenosis. One viable solution to solve this complication is using drug eluting stents (DES) (Liu et al., 2014; Condello et al., 2023). DES are a time-ordered drug release; these types of stents are made of stainless steel, nickel-titanium, and cobalt-chromium and coated with biodegradable polymers to achieve loading drugs (see Table 1) (Borhani et al., 2018). Even though DES has uncountable advantages over stents, concerns remain about the long-term clinical application of DES. One of the new alternatives to DES is shaping memory stents and polymer-free drug-eluting stents (Tigkiropoulos et al., 2022). On the other hand, when the artery is completely blocked by atherosclerotic plaque or the stent fails, surgical alternatives still exist to solve this kind of problem. One possible solution is redirecting the blood flow from one area to another by reconnecting blood vessels (artery, vein, capillary). This redirection and reconnection of the blood flow can be achieved with vascular grafts (autografts or synthetic) (Martínez-González et al., 2017; Shahriari-Khalaji et al., 2023).

**TABLE 1:** Materials involved in the development of CMDs to solve cardiac anomalies.

Disease	Medical device	Employed materials	References
Valvular heart disease	Surgical bioprosthetic valves	Biological components (pig or bovine)	Arcidiacono et al. (2005), Abbasi et al. (2019)
		Chemical compounds (glutaraldehyde)	
	Mechanical heart valves	Metals (Co-Cr alloys, Ti alloys, nitinol frames)	Kütting et al. (2011)
		Polymers (polyester, thermoplastic polyurethanes)	
		Other materials (pyrolytic carbon)	
	Percutaneous transcatheter valves	*Mitral clip*	Panaich and Eleid (2018), MitraClip (2022)
		Metals (Cr-Co)	
		Polymers (polyester)	
Coronary artery disease	Stents	*Self-expandable*	Korei et al. (2022), Shen et al. (2022)
		Metals (SS, Pt-iridium, Cr-Co)	
		*Shape memory materials*	
		Metals (nitinol alloys)	
	Drug eluting stents	Metals (SS, nitinol, and Co-Cr)	Borhani et al. (2018)
		Polymers (polylactic acid, poly-L-lactic acid, poly(lactic-co-glycolic acid), poly(DL-lactide))	
	Vascular grafts	*Synthetic vascular grafts*	Shahriari-Khalaji et al. (2023)
		Polymers (polyethylene terephthalate and expanded polytetrafluoroethylene)	
		*Autografts*	Martínez-González et al. (2017)
		Biological components (autologous veins)	
Cardiac arrhythmias	Cardiac pacemakers	Metals (Ti alloys, Ni-Co-Cr-Mo alloy (MP-35N) and Pt	Gradaus et al. (2003), Medtronic (2017)
		Polymers (silicone, polyurethane)	
	Implantable cardioverter-defibrillators	Metals (Ti alloy, Ni-Co-Cr-Mo alloy (MP-35N) and Pt	
		Polymers (silicone, polyurethane, fluoropolymers)	
Congestive heart failure	CardioMEMS HF systems	Polymers (Silica and silicone)	Ayyadurai et al. (2019)
	Cardiopulmonary bypass	*Extracorporeal membrane oxygenation (ECMO)*	Torregrosa et al. (2009), Sultan et al. (2018)
		Polymers (polypropylene, silicone)	
		*Mini extracorporeal circuit (MECC)*	
		Polymers (polypropylene, silicone)	
		Chemical compounds (heparin poly-2-methoxyethyl acrylate, synthetic protein, phosphorylcholine)	
	Ventricular assistant devices	*Intraaortic balloon pump (IABP)*	Meyer et al. (2011), Valdovinos (2013), Sultan et al. (2018), Abiomed (2022), Karimov et al. (2020)
		Polymers (polyethylene, polyurethane)	
		*TandemHeart*	
		Metals (SS, Cu)	
		Polymers (polyurethane)	
		*Impella device*	
		Polymers (silicone, polyurethane)	
		Metals (platinum, nitinol)	
		*VA-ECMO*	
		Polymers (polyurethane, polyvinyl chloride)	
		Metals (SS)	
		Other materials (polymethyl pentene fibers)	
	Ventricular assistant devices and Total artificial heart	***Pulsatile flow***	Ufukerbulutt and Lazoglu (2011), Slepian et al. (2013), Valdovinos (2013), Ahmed et al. (2020), Karimov et al. (2020)
		*Abiomed BVS 5000i (Abiomed Cardiovascular, Inc., Danvers, MA)*	
		Polymers (polyurethane)	
		*Thoratec (Thoratec Corporation, Pleasanton, CA) (paracorporeal VAD)*	
		Polymers (thoralon polyurethane, polycarbonate, polyester velour)	
		Metals (SS)	
		Other materials (pyrolytic carbon, delrin)	
		*Berlin Heart (German Heart Institute, Berlin)*	
		Polymers (polyurethane, silicon)	
		Other materials (dacron-velour)	
		***Pulsatile-intracorporeal devices***	
		*Thoratec HeartMate XVE LVAS (Thoratec Corp., Pleasanton, CA)*	
		Polymers (polyurethane)	
		Metals (Ti alloy)	
		Other materials (porcine xenograft, woven Dacron)	
		*Novacor (LVAS) (World Heart Corp., Oakland, CA)*	
		Polymers (polyurethane, polytetrafluoroethylene)	
		*Thoratec*	
		Polymers (Thoralon polyurethane, polyester velour)	
		Metals (Ti alloy)	
		Other materials (pyrolytic carbon, delrin)	
		***Continuous axial flow***	
		*Biomedicus Bio-Pump (Medtronic BioMedicus, Inc.)*	
		Polymers (polycarbonate)	
		*Sarns centrifugal pump (3-M Health Care, Ann Arbor, MI)*	
		Polymers (polycarbonate acrylic, silicone rubber, nylon)	
		Metals (aluminium oxide)	
		*Evaheart (Eveheart Medical USA, Inc.) (long-term support)*	
		Polymers (polytetrafluoroethylene, silicon carbide, polyester)	
		Metals (Ti)	
		Other materials (carbon graphite)	
		*Gyro Centrifugal*	
		Polymers (polycarbonate, polyethylene, polyurethane polyvinyl, polyvinylchloride)	
		Metals (SS, Ti)	
		Other materials (alumina ceramic)	
		*MicroMed DeBakey VAD (MicroMed Cardiovascular Inc., Houston, TX)*	
		Metals (polished Ti)	
		Other materials (Dacron)	
		*Jarvik-2000 (Jarvik Heart Inc., New York, NY)*	
		Metals (Ti)	
		Other materials (ceramic)	
		*Thoratec HeartMate II LVAD (Thoratec Corp., Pleasanton, CA)*	
		Polymers (polypropylene, dracon)	
		Metals (Ti)	
		***Third generation of blood pumps***	
		*Incor (BerlinHeart, German Heart Institute, Berlin)*	
		Polymers (silicon)	
		Metals (Ti alloy)	
		*Ventrassist (Ventrassist Division, Ventracor Ltd, Chatswood, NSW, Australia)*	
		Polymers (silicone, polyester)	
		Metals (Ti, Al-V alloy)	
		*HeartMate III (Thermo Cardiosystems Inc.)*	
		Polymers (polyester)	
		Metals (Ti)	
		***Total artificial hearts***	
		*CardioWest (SynCardia Systems Inc., Tucson, AZ)*	
		Polymers (polyurethane, PVC, dracon)	
		Metals (Ti alloys)	
		AbioCor (Abiomed Cardiovascular Inc., Danvers, MA)	
		Polymers (polyurethane, dracon)	
		Other materials (stycast epoxy)	
Atrial septal defects	Closure devices	Polymers (polytetrafluoroethylene)	Padera and Schoen (2020)
		Metals (nitinol, platinum)	
Atrial Fibrillation	Left atrial appendage occlusion devices	Metals (nitinol, Ti)	Padera and Schoen (2020)
		Polymers (polyethylene terephthalate)	

Abbreviations: LVAD, left ventricular assistant devices; SS, stainless steel; TAH, total artificial heart, PVC, polyvinyl chloride.

Another cardiac pathology is valve problems. The intracardiac valves (mitral, tricuspid, pulmonary, and aortic) are critical in assuring unidirectional forward blood flow through the myocardium (Sacks et al., 2009). However, when one of these valves is compromised, the heart function is affected. It leads to either stenosis (obstruction to flow) or regurgitation (reverse flow across the valve) (Powers and McCulloch, 2022). Besides, these two problems can occur in the same valve (Camm et al., 2018). One way to solve valvular problems is by repairing the existing abnormal valve tissue to make it functional (Fedak et al., 2008). However, when a repair is not an option, CMDs is intended to bring light to valve problems. CMDs employed to solve valve problems are divided into two categories i) surgical implantation, which include bioprosthetic and mechanical valves (Karimov et al., 2020), and ii) transcatheter aortic valve implantation (TAVI) (Khan and Alam, 2022). Materials used to develop CMDs for valvular heart diseases are presented in Table 1.

Cardiac arrhythmias are defined as anomalies or disturbances present in the cycle of the cardiac electrical rhythm (Fu, 2015; Vicent and Martínez-Sellés, 2021). Cardiac arrhythmias can be treated with surgical procedures that include cardiac ablation (McBride et al., 2021). When cardiac ablation is not a viable option, cardiac arrhythmias can be treated with CMDs, for instance, implantable cardiac defibrillators and pacemakers. Cardiac pacemakers are made of metals, Ti, and MP-35N alloys, principally (see Table 1) (Gradaus et al., 2003; Medtronic, 2017). Cardiac pacemakers involve long-term implantation, for this reason, the chosen materials must be of excellent strength and corrosion resistant. On the other hand, ICDs are made of metals (e.g., Ti, MP-35N, and Pt alloys) and polymers (e.g., silicone, polyurethane, and fluoropolymers) (Gradaus et al., 2003; Medtronic, 2017). In order to ensure the ICDs usefulness, the materials must be carefully chosen and, most importantly, resistant to corrosion. However, ICDs have a lifespan of 3–5 years, which corresponds to a much shorter lifespan compared to pacemakers (Padera and Schoen, 2020).

On the other hand, congestive heart failure (CHF) is the result of a deficiency of pump (Truby and Rogers, 2020). CHF affects more than 6.2 million people in the USA, with a higher incidence over the age of 65 (Kepinska et al., 2019). The CMDs employed to solve CHF are mainly classified into three fields, 1) percutaneous ventricular assist devices or short-term mechanical circulatory supports, 2) durable ventricular assist devices, and 3) total artificial hearts (Han et al., 2018). A summary of materials used to develop CMDs for CHF is presented in Table 1.

Atrial septal defects (ASD) fall under the classification of congenital heart anomalies. A secundum atrial septal defect is a gap in the wall that connects the heart’s two upper chambers and is the most common type of ASD. This passageway allows blood to flow between the upper chambers, increasing blood flow to the lungs. There are five main devices develop to solve ASD, of which only two have been approved by the Food and Drug Administration (FDA); Amplatzer^®^ septal occluder and GORE-HELEX^®^ septal occluder (Geva et al., 2014). The remaining devices that have only been approved by the Conformité Européenne (CE) are Figulla atrial septal defect occluder, Cera atrial septal defect occlude, and Ultrasept atrial septal defect occluder (Geva et al., 2014). The materials (see Table 1) for construction for these devices need to be mechanically resistant and flexible. For example, the materials employed in the development of atrial septal defects are made of metals (e.g., nitinol and platinum) and polymers (such as polytetrafluoroethylene) (Padera and Schoen, 2020; Vishwanath et al., 2022).

Another cardiac pathology is atrial fibrillation (AF). AF is a common heart rhythm disorder with an estimated 33 million people affected worldwide (Wijesurendra and Casadei, 2019), characterized by rapid and uncoordinated atrial electrical activity (Zimetbaum, 2017). Due to this lack of coordination, the upper chambers of the heart quiver or fibrillate, resulting in poor contractile function and irregular flow inside the chamber (Lau et al., 2019). One of the greatest concerns in AF is cardiovascular stroke since they are related to thrombus formation since the left atrial appendage (LAA) is a common site of thrombus formation (Beigel et al., 2014; Kallistratos et al., 2018). Due to this, specialized devices for this cardiac anomaly are intended to solve problems with left atrial appendage occlusion (LAAO) disease. Among the current devices, we can highlight that only two devices out of seven are approved by the FDA: Lariat device and Watchman (Meier et al., 2018; Price, 2018; Pacha et al., 2019; Obeid et al., 2020; Wintgens et al., 2020; Jackson et al., 2021). In order to develop cardiac medical devices to solve LAAO, the materials need to be flexible, durable, and also mechanical resistant (see Table 1). Moreover, not only the material selection is a critical step in the developing process of CMDs also biocompatibility and hemocompatibility are main concerns.

## 3 Problems in cardiac medical devices

Materials used in CMDs are often called biomaterials and have specific design criteria, especially in applications where the device contacts, is temporarily inserted or is permanently implanted in the body (see Section 2). Biomaterials have to achieve biocompatibility standards as nontoxic, non-thrombogenic, and noncarcinogenic standards, neither mutagenic nor antigenic. These biocompatibility standards largely determine the interaction between the materials and proteins in the biological environment interactions with tissues and cells.

For instance, once the CMD is implanted, multiple factors can interfere with or inactivate the CMDs function: inflammation, coagulation, platelet adhesion, and thromboresistance (Helmus et al., 2008; Qi et al., 2013; Ghasemi-Mobarakeh et al., 2019). The interactions between the CMDs surface and redundant medium determine the biocompatibility and the hemocompatibility (Chen and Wang, 2013; Dharavath and Maddi, 2022).

**FIGURE 1:**
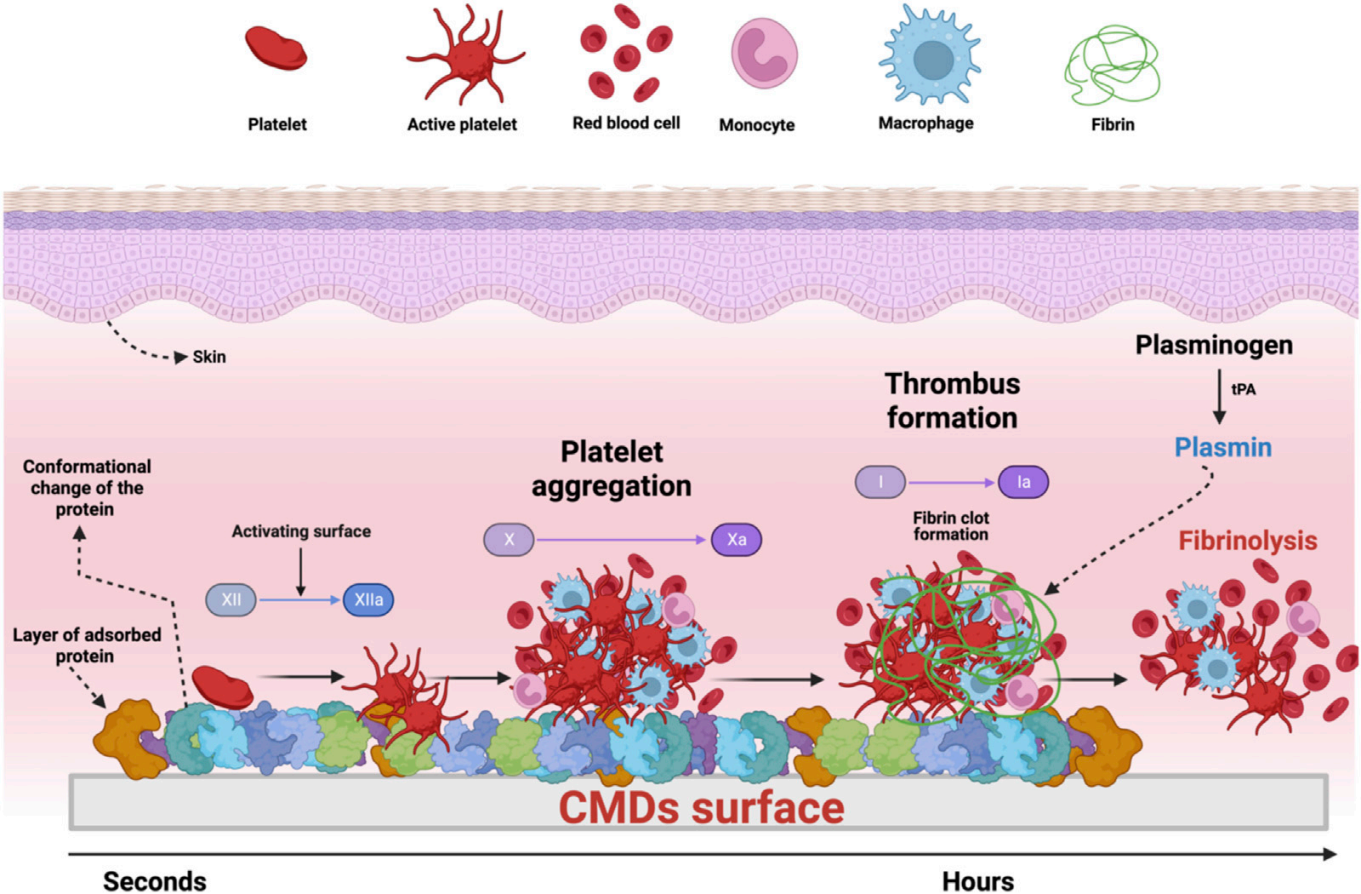
Interaction of human blood with CMDs. When the biocompatibility of the CMD is low, plasma proteins are adsorbed on the CMD surface. This event led the activation of the coagulation factor XII and activate the intrinsic pathway of the coagulation cascade. Consequently, the thrombus formation take place with the aggregation and fibrin clot formation (Factor I to factor I activated). Plasmin is the final product of the activation of plasminogen with the tissue plasminogen activator (tPA) and degrades fibrin, allowing the restoration of the continuous blood flow in vessels.

The first interaction between CMDs and the redundant medium is plasma proteins. Plasma proteins are the most significant components of blood and can be adsorbed on the CMDs surface in a matter of a few seconds (see Figure 1) (Schenone et al., 2004; Rabe et al., 2011). Once the plasma proteins are attached to the material surface, the adsorbed proteins change their tridimensional conformation and expose their active site. All the events mentioned above address to inflammatory response and activation of the coagulation cascade. These conformation changes lead to the activation of coagulation factor XII and activate the intrinsic pathway of the coagulation cascade (Schenone et al., 2004; Chen et al., 2007; Vogler and Siedlecki, 2009; Golas et al., 2010; Chernyshenko et al., 2022). Afterward, platelets respond to several external stimuli (e.g., thrombin from the coagulation cascade, adenosine diphosphate released from destroyed cells, and shear force) (Schenone et al., 2004; Chernyshenko et al., 2022). These external events stimuli conduct the adhesion, activation, and aggregation of platelets at any thrombogenic surface (Mackman, 2008; Freitas et al., 2010). Platelet activation leads to CMDs failure by forming a stable thrombus, an occlusive and lumen narrowing complex with fibrin incorporated in the growing platelets plug. Besides platelets and fibrinogen deformation, thrombin activation is critical in the common coagulation pathway and feedback loops. It acts as a protease that converts soluble fibrinogen into fibrin, polymerizing into insoluble clots. Thrombin also catalyzes other coagulation-related reactions, such as amplifying the Factor XIa conversion from Factor XI in the extrinsic system (Gorbet and Sefton, 2004; Mackman, 2008). The complement activation and associated inflammatory reactions occur in parallel, playing the dominant role in the body’s defense against foreign materials (see Figure 1) (Ferrari and Peyvandi, 2020; Chen et al., 2022). Non-biocompatible CMDs present a series of unfavorable reactions, protein adsorption on the surface of the materials, initiation of the coagulation cascade, platelet activation, fibrin formation, inflammation, etc. These reactions lead to rejection by the host (Suarez-Pierre and Kilic, 2019). The biological response to biomaterials is a crucial concern in the design of CMDs. Moreover, CMDs are made of multiple materials (see Table 1), and the choice of the best biocompatibility material needs to be sacrificed for other characteristics (e.g., stiffness, strength, corrosion resistance, hardness, and rigidity), which allows the CMDs to have an excellent workflow (see Section 2).

On the other hand, not only the biocompatibility and hemocompatibility is a critical point, infections in CMDs have become extremely relevant in recent decades. The predominance of bacterial infection on CMDs such as cardiac implantable electronic devices, VAD, and stents have increased considerably (Deter, 2000; Refaat et al., 2019; Toriello et al., 2022; Zijderhand et al., 2022). *Staphylococcus* species is one of the main responsible of infections in surgical procedures (Bongiorni et al., 2012). However, infections with the following species have also been reported: *Corynebacterium spp, Propionibacterium spp,* Enterobacteriaceae species, *Pseudomonas* spp., *Candida albicans*, *Mycobacterium abscessus* and *Aspergillus fumigatus* (Alessandro et al., 2004; Bongiorni et al., 2012). Infections predispose the patient to complicated surgical interventions or abrasive treatments with antibiotics (Comba et al., 2022). Due to this, the need arises to formulate biocompatibility strategies, including the ability to avoid infections by pathogenic agents.

One viable solution to enhance the biocompatibility and hemocompatibility of the CMDs is the implementation of surface modifications. Surface modifications allow the immobilization of bioactive compounds or chemical molecules onto surfaces. For instance, surface modifications to enhance biocompatibility and hemocompatibility include: passive surface, bioactive surface, and endothelization. Moreover, these surface modifications only allow solving biocompatibility/hemocompatibility issues (e.g., platelet activation, inflammatory response, coagulation pathways, complement activation, and immune response). On the other hand, not only the biocompatibility and hemocompatibility issues can be addressed with surface modifications. Infections also can be addressed. This type of surface modification allows to avoid infections with the use of hydrogels, antibiotics bags, and, recently with, antimicrobial peptides. (Nie et al., 2016; Yang et al., 2018; Kazemzadeh-Narbat et al., 2021; Sánchez-Bodón et al., 2022). Surface modifications are the golden key to achieve the future generation of CMDs.

## 4 Surface modifications in cardiac medical devices

Besides the properties of the materials employed in the development process of CMDs (e.g., stiffness, strength, corrosion resistance, hardness, and rigidity), the correct selection of materials is essential when the goal is increasing the device’s biocompatibility. The surface modification allows the binding of chemical or bioactive compounds to the surface of the blood-contacting material. Several coatings strategies have been developed through the years, and some of them are already commercially used in CMDs. A good interaction between the device and the blood will determine the device’s success in the short or long term (Mozafari, 2020). An excellent hemocompatibility will prevent reimplantation, avoiding possible infections related to the manipulation of the device.

### 4.1 Passive surfaces

Passive surfaces are strategies to avoid protein adsorption and blood components to the surface of CMDs. When a protein is irreversibly adsorbed on the surface, it will lead to platelet activation and finally will end in the failure of the CMDs (see Figure 1). To avoid protein adsorption, passivated surfaces (i.e., with inorganic coatings) with modified interfacial energy will avoid protein adsorption (He et al., 2008; Zhang et al., 2008). Instead, organic coatings can also be used by creating super-hydrophilic surfaces with repellent surface charges and layering hydrophilic macromolecules to take advantage of hydration forces and steric hindrance, giving them the ability to avoid protein adsorption (Brynda et al., 2000; Rodriguez-Emmenegger et al., 2011; Erathodiyil et al., 2020). The development of CMDs blood compatibility can use both inorganic and organic coatings (Sarkar et al., 2012; van de Wouw and Joles, 2022). Finally, the reversible protein adsorption onto the blood-contacting material due to the textured surface represents a suitable option to prevent conformational changes in proteins that lead to platelet activation (Riveiro et al., 2018).

#### 4.1.1 Inorganic coatings

**FIGURE 2:**
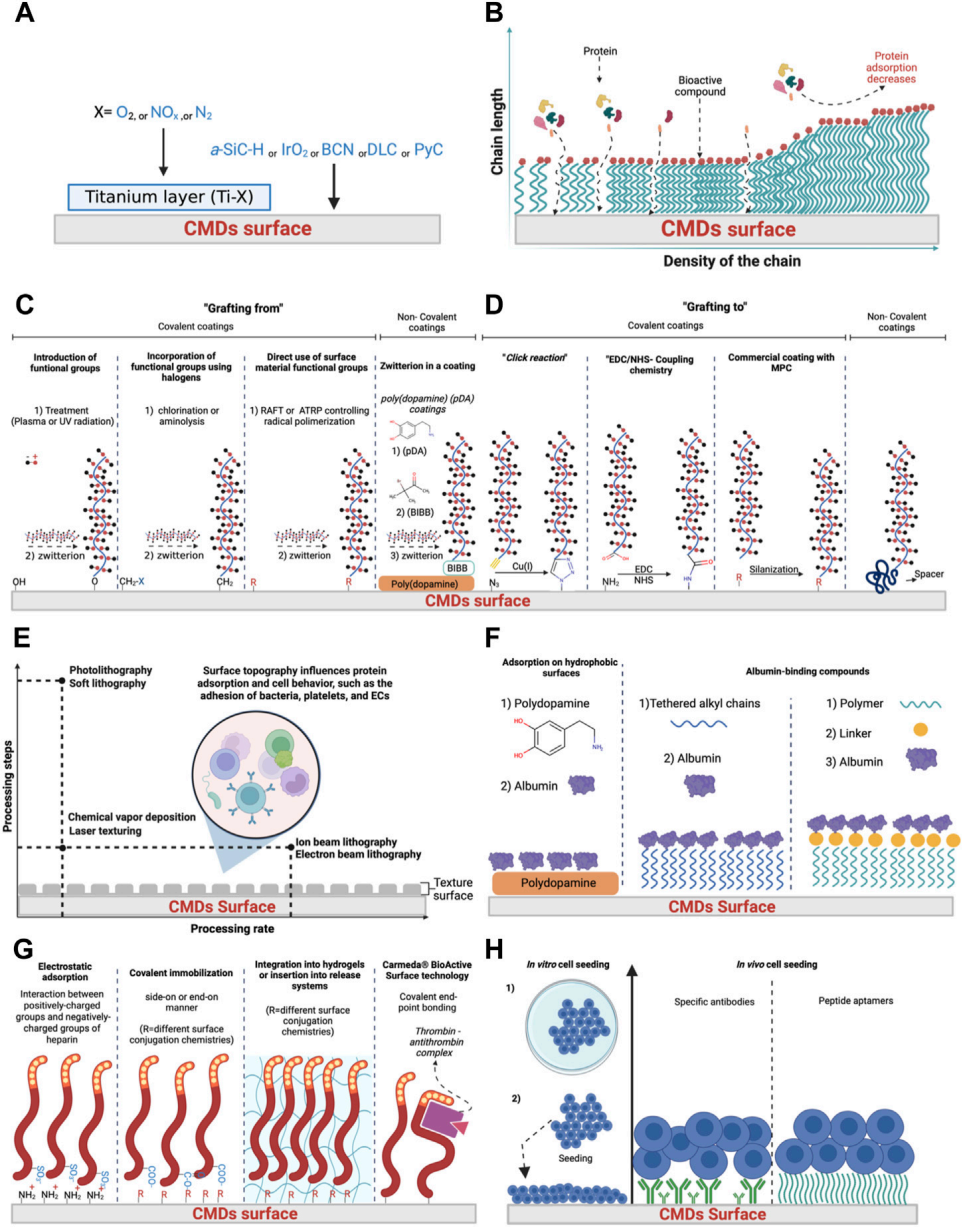
Surface modification of blood-contacting materials. Inorganic surfaces **(A)**, brush forming polymers **(B)**, zwitterions polymers **(C,D)**, texture surface **(E)** albumin coatings **(F)**, anticoagulant surfaces **(G)**, and endothelization **(H)**. Abbreviations. A-SiC-H = amorphous silicon carbide, BCN = boron-carbon-nitrogen, DLC = diamond like carbon, PyC = pyrolytic carbon, RAFT = reversible addition-fragmentation polymerization, ATRP = atom transfer radical polymerization, BIBB = 2-Bromo-2-methylpropanoyl bromide, MPC = 2-methacryloyloxyethyl phosphorylcholine, ECs = endothelial cells.

In the domain of blood-contacting biomaterials, inorganic coatings (metal oxides, metal nitrides, and carbon-based coatings) (see Figure 2A **)** are the most studied. Inorganic coatings generally have a high level of inertness, and mechanical and chemical stability. They are primarily used for passive hemocompatible coatings and corrosion protection (Werner et al., 2007). Metals, such as Ti and Ti-alloys are mostly used in the development of CMDs (e.g., stents, artificial heart valves, and blood pumps) due to their exceptional biocompatibility (Werner et al., 2007; Kuchinka et al., 2021). Coatings with metal oxides and metal nitrides avoid protein adsorption; for instance, titanium oxide as a coating exhibit different surface energy, allowing to have good hydrophobicity and low protein adsorption (Werner et al., 2007). Also, it has been reported the inorganic coating depends on its thickness, with thicker films becoming much more hemocompatible (Nygren et al., 1997; Fischer et al., 2018). On the other hand, metal nitrides on titanium surfaces (TiN), effectively prevent thrombus formation despite their lower performance in wear resistance (Dioni et al., 1992). The hemocompatibility properties of inorganic coatings led to the implementation of metals nitrides on titanium surfaces (TiN) of CMDs (e.g., heart valves, heart assist devices, and heart pumps) (Dioni et al., 1992; Wan et al., 2005; Sin et al., 2012). These layers of TiO and TiN can be produced by physical or chemical vapor deposition, and film deposition (Wan et al., 2005; Werner et al., 2007).

Another inorganic modification designed to improve hemocompatibility is diamond-like carbon (DLC) coating. DLC is a well-established coating for improving the hemocompatibility of artificial heart valves, vascular stents, and ventricular assist devices (Werner et al., 2007; Fedel et al., 2009). It can be used for metals and polymers (Alanazi et al., 2000; Krishnan et al., 2002; Dearnaley and Arps, 2005). This coating has high strength and smoothness, chemical inertness, minimal wear, and a low frictional coefficient (Fischer et al., 2018). DLC contains both types of carbon-carbon bonds, near-planar trigonal (sp2) and tetragonal (sp3). It has been reported that DLC coatings can be more rigid and brittle when the sp3:sp2 ratio is increased (Dearnaley and Arps, 2005). Despite their good properties, DLC coatings have some limitations, including the formation of micro-cracks. It has been reported these micro-cracks promote thrombus formation and carbide formation when in contact with iron (Werner et al., 2007; Sin et al., 2012). To overcome these limitations, several methods have been reported to create DLC coatings, including chemical vapor deposition, pulsed laser deposition, direct ion beam deposition, cathodic arc deposition, magnetron sputtering, and plasma source ion deposition (Dearnaley and Arps, 2005). Another alternative to inorganic coatings is boron-carbon-nitrogen and pyrolytic carbon (PyC) film, with similar mechanical and biological properties of DLC (More et al., 2013). These alternative coatings are created through chemical vapor deposition. Also, it has been reported and tested on CMDs such as stents and artificial heart valves (Maitz et al., 2006). Boron-carbon-nitrogen coating and PyC represent a good option when short-time is required because it has been reported in long-term applications shown platelet adhesion (see Table 2) (Kim et al., 2005; Sick et al., 2005; Sarkar et al., 2012).

**TABLE 2:** Advantages and disadvantages of surface modification techniques.

Surface modification	Generalities	Specific methods to develop a coating	Advantages	Disadvantages	References
Inorganic coatings	High level of inertness and mechanical and chemical stability	Deposition methods	Exhibit different surface energy, allowing to have good hydrophobicity and low protein adsorption	Mainly applied to metal surfaces and rarely to ceramics due to the low attachment of inorganic molecules onto ceramic surfaces	Werner et al. (2007)
			DLC coating has high strength and smoothness, chemical inertness, minimal wear, and low frictional coefficient	Carbide formation in contact with iron and micro-cracks forming on the surface (promotes thrombus formation)	Werner et al. (2007), Hedayati et al. (2019)
			Boron- carbon-nitrogen and pyrolitic carbon film. Better alternative than DLC coatings	Platelet adhesion in long-term applications	Hedayati et al. (2019)
			Amorphous silicon carbide (a-SiC:H). Similar properties than metal oxides, metal nitrides and oxide nitrides	NR	Werner et al. (2007)
			Iridium oxide coating could represent a better option in terms of inorganic coatings for ICDs due to the low-polarization material. IrO_2_ have shown a decrease in electrode polarization as well as a reduction in pacing thresholds	NR	Werner et al. (2007)
Brush-forming polymers	Regulate protein surface and cell surface interactions	Grafting from	Achieved higher grafting densities and film thicknesses	Chemical stability decreases in the presence of metal ions and oxygen which significantly reduces their antifouling properties	Hedayati et al. (2019)
		Grafting to		Methods are restricted because of steric hindrance amongst the polymer chains that makes it complicated to tether chain ends at short intermolecular distances	Murad Bhayo et al. (2022)
Zwitterionic polymers	Attempting to prevent protein adsorption	Grafting from	Several techniques and they depend on the surface of the material	Taking mechanical properties into account, these polymer coatings are not as strong or stable as the DLC or Ti–O film	Qi et al. (2013)
		Grafting to	Most studied due to high yield of attachment product		
				Polymer anti-coagulant coatings alone seem not to be an ideal for long term implant but a good choice for short term application	
Passive albumin layer	Reduced platelet reactivity	Adsorption on hydrophobic surfaces	Rapid method to immobilize albumin	Mainly designed for polymeric materials and short-term devices	Barberi and Spriano (2021)
		Surfaces are reversibly immobilized with albumin	Avoid albumin denaturalization		
				Albumin will suffer denaturation and degradation or be displayed by other proteins if it does not choose the correct attachment chemistry	
Texture surfaces	Improving the hemocompatibility of blood-contacting biomaterials	Photolithography, soft lithography, chemical vapor deposition, laser texturing, atomic force microscopy, ion beam lithography, and electron beam lithography	Must be selected in accordance with the needs and time	Not all the techniques can be made with less steps. Some of them waste several chemicals in the process. Also, not all the techniques can work with large areas to treat. And finally, some of them involves high cost of production	Riveiro et al. (2018)
Anticoagulant surfaces	Avoid activation of the coagulation cascade	Electrostatic absorption, covalent immobilization, and recently integration into hydrogels or insertion into release systems	Produce an effective thrombin-antithrombin complex	Heparin coatings with the structural changes caused by immobilization reactions may inactivate covalently bound heparin	Fischer et al. (2018)
				Heparin is manly replaced with analogues, another anticoagulant protein and contact system specific inhibitor	
				Short half-time of the heparin	
Platelet inhibitors	Avoid the activation of platelet formation as well coagulation factors and inflammatory reactions	Immobilization of enzymes, chemical compounds, and prostaglandins	Rapid method to inhibit receptors or cycles that will led to platelet aggregation	The chemical compound that is attached can suffer degradation or inactivation	Liu et al. (2014b), Yang et al. (2015)
		Immobilization of NO or realizing NO	Aims to mimic the natural way of healthy blood vessels to inhibit platelet aggregation	NO release does not confer efficient long-lasting anti-thrombogenicity on the surface material. Other functions may be necessary, such as those provided by active anticoagulants or antiplatelet inhibitors	
Fibrinolytic agents	Preventing thrombus formation, including the activation of blood platelets	Immobilized tPA or capturing tPA for the blood stream	Thrombus formation is avoided	This technique must be more investigated. Possible conjugations with clinically approved plasminogen activators	Hedayati et al. (2019)
Endothelialization	Promote endotheliazation to avoid rejection of the foreign material	*In vitro* cell seeding, subsequently seeded	Allows to grow EC outside the patient, with efficient control environments	*In vitro* EC seeding is a very time-consuming and cost-intensive procedure, and is challenging because of problems with cell sourcing, cell stability, and cell viability	Fischer et al. (2018), Hedayati et al. (2019)
				Moreover, in the case of autologous cells, there is the need of two operative procedures increasing the possibility of contamination and infection. Additionally, the use of allogenic cells can cause rejection	
		*In vivo* self-endothelialization	Allow grow EC inside of the patient	The method is not very selective, so that in addition to ECs, other cells can also grow on the surface	
Antimicrobial coatings	Avoid bacterial infections	Antibiotics attachment	Bacterial infections can be treated	The effectiveness depends on several factors (concentration, release mechanism, and bacterial resistant)	Riool et al. (2017)
		AMPs attachment	Avoid, fungal, bacterial and virus infections. Apport a wide spectrum of use	Need to optimize the thickness, density and high in order enhance their activity. More research must be applied on this technique	

Abbreviations: NR, no reported; DLC, diamond like carbon; NO, nitric oxygen; tPA, tissue-type plasminogen activator; AMPs, antimicrobial peptides; EC, endothelial cells.

One commercial technique that recently attracted attention is the amorphous silicon carbide (a-SiC: H). a-SiC-H has semiconductor and analogs properties to TiO. a-SiC-H is employed as a coating on vascular stents and heart valves (Werner et al., 2007; Knaack et al., 2016; Saddow, 2022). It is important to highlight one inorganic coating which is still in clinical studies, iridium oxide (IrO_2_), has shown good biocompatibility and increased performance in vascular stents. IrO_2_ coating could represent a better option in terms of inorganic coatings (Niebauer et al., 1997; Werner et al., 2007).

#### 4.1.2 Organic coatings

Organic coatings such as polymers brushes, and zwitterion polymers create super-hydrophilic surfaces with repellent surface charges and layering hydrophilic macromolecules to take advantage of hydration forces and steric hindrance, giving them the ability to avoid protein adsorption. Several methods have been developed through the years to enhance their efficiency and overcome their disadvantages (“grafting to,” “grafting from”) (Brynda et al., 2000; Rodriguez-Emmenegger et al., 2011; Erathodiyil et al., 2020).

##### 4.1.2.1 Polymers brushes

Polymer brushes represent a novel surface modification with characteristics that regulate protein-surface and cell-surface interactions (Brittain and Minko, 2007; Chen et al., 2017; Hucknall et al., 2009; Moroni et al., 2014). Polymer brushes coatings are polymer chains anchored to the CMDs surface. Once the polymer chain is anchored, it can adopt different conformations depending on the density and molecular weight of polymer chains (Murad et al., 2022). The length and density of immobilized macromolecules significantly affect protein adsorption, which means those proteins with low molecular weights and small sizes, may still find adsorption sites if the surface coating is not dense enough (see Figure 2B). In the field of CMDs, polyethylene glycol (PEG) is the most researched compound in hydrophilic brush-forming polymers (Advincula, 2004; Riedel et al., 2013; Murad Bhayo et al., 2022). The steric repulsion force is the main effect of PEG surfaces. The PEG chains can form a hydration layer, forming a hydration barrier between the surface and the blood that resists the adsorption of plasma proteins to some extent. Besides the good properties of PEG, PEG brushes are not appropriate for long-term use because of metal ion-catalyzed oxidation, which can lead to the decomposition of the PEG brushes (Andersen et al., 2013; Riedel et al., 2013; Hedayati et al., 2019; Ashcraft et al., 2021; Murad Bhayo et al., 2022).

Polymer brushes can be synthesized using one of two methods: “grafting to” or “grafting from.” “Grafting to” method is less employed because it is not possible to achieve high grafting densities. Low grafting densities are due to steric hindrance amongst the polymer chains, which makes it challenging to tether chain ends at short intermolecular distances (see Figure 2B). Furthermore, brush thickness achieved by “grafting to” methods are defined and limited by the molar mass of the polymers, which represents another complication of this method (Mocny and Klok, 2020). On the other hand, “grafting from” is more frequently used due to higher grafting densities and film thicknesses. Additionally, “grafting from” uses bottom-up strategies that involve the formation of polymer chains via surface-initiated polymerization from a substrate modified with functional groups capable of initiating polymerization (Murad Bhayo et al., 2022). It has been reported different methods for surface-initiated polymerization which can be used to develop polymer brushes. These include both uncontrolled and controlled strategies: 1) free radical polymerization, 2) reversible addition-fragmentation polymerization (RAFT), 3) nitroxide-mediated radical polymerization (NMP), 4) ionic polymerization, and 5) ring-opening metathesis polymerization (ROMP) (Whiting et al., 2005)

Polymer brushes described previously can be optimized (e.g., density, thickness, hydrophobicity, height, weight) to inhibit nonspecific protein adsorption and subsequent fouling from whole blood. Thus, chemical stability tends to decrease in the presence of metal ions and oxygen, lowering their antifouling properties significantly (Hedayati et al., 2019).

To be applied to CMDs for short or long-term use, it has been reported polymers brushes with modified polymers such as poly (methacrylates), poly (acrylamides), and poly (2-oxazolines), which are suitable for multiple potential applications. For the moment, these modified polymers are used in the preparatory work of protein-resistant and blood-compatible polymer brushes (Adams and Schubert, 2007; Hoogenboom, 2009; Rodriguez-Emmenegger et al., 2011; Riedel et al., 2013; Surman et al., 2015; Harris et al., 2019).

##### 4.1.2.2 Zwitterionic polymers

To prevent protein adsorption, zwitterionic interfaces that contain anionic and cationic groups and have an overall neutral charge are an appealing option for polymer brushes (Estephan et al., 2011). (see Figure 2C) (Qi et al., 2013; Shao and Jiang, 2015). Due to their excellent anti-fouling properties, zwitterionic polymers have recently demonstrated great potential in anti-coagulation coating. Also, it has been reported that the electrostatic interactions can develop a hydration layer on the surface of zwitterionic polymers, increasing their resistance to non-specific protein adsorption, similar to PEG surfaces or other types of polymer brushes (Estephan et al., 2011). Coatings with zwitterionic properties are a promising strategy for increasing the hemocompatibility of VADs, stents, and oxygenators (Erathodiyil et al., 2020).

There are two general methods to develop zwitterionic coatings, “grafting to” and “grafting from.” “Grafting from,” the most common technique, can be divided into four categories: 1) plasma processing; used to incorporate hydroxyl groups onto the material surface), 2) incorporation of new functional groups on the surface using halogens. Based on the type of attachment employed, allowing for the direct modification of the selected surface, chemical treatments have to be employed for each surface type (e.g., chlorination or aminolysis), 3) direct use of the surface material functional groups; method of choice for cellulose membranes, and iv) mussel-inspired poly (dopamine) (pDA) non-covalent coatings (see Figure 2C). On the other hand, “grafting to” refers to polymers that are attached to the surface as the last step (Semak et al., 2017). “Grafting to” allows to perform of several chemistry reactions such as click-reaction, EDC/NHS coupling chemistry, and non-covalent reactions (see Figure 2D).

The most thoroughly studied classes of zwitterionic materials are betaines, carboxybetaines, sulfobetaines, polyampholytes, and phosphorylcholines (He et al., 2008; Nakabayashi and Williams, 2003; Smith et al., 2012; Yang et al., 2009; 2016; Zhang et al., 2013). Among all zwitterion structures created for blood contact applications, phosphorylcholine is a promising compound since prompted the development of a new class of phosphorylcholine polymers called 2-methacryloyloxyethyl phosphorylcholine (MPC) (see Figure 2D) (Nakabayashi and Williams, 2003). The amount of protein attaching to blood-contacting material is influenced by the surface’s free-water fraction; surfaces with a high free-water fraction have minor protein adsorption. MPC coatings retain a high free-water fraction, and the phosphorylcholine groups assist in lowering protein adsorption at the interface (Badv et al., 2020). Protein adsorption, platelet adhesion, and complement activation have all been shown to be reduced on surfaces with immobilized MPC (He et al., 2008; Schlenoff, 2014). It has been reported that despite the good properties of MPC polymers, their antithrombotic property in MPC-blood pumps could have a limited lifetime, implying that an anticoagulant will still be considered necessary after implantation (Nakabayashi and Williams, 2003; Shao and Jiang, 2015).

On the other hand, a combination of PEG and zwitterionic compounds could enhance the blood-contacting material’s overall blood compatibility, and several studies have been reported (Lee et al., 2011). Also, the high anti-fouling ability is disadvantageous to specific cell adhesion and growth in zwitterionic coatings using polycarboxybetaine functionalized with peptide (REDV) through functionalization of carboxy groups in carboxybetaine by NHS/EDC chemistry previously reviewed to improve selectivity for endothelial cells (see Table 2 and Figure 2D) (Ji et al., 2012).

Furthermore, the formation of an endothelial layer on the surface of CMDs is essential for antithrombosis and is a challenge for permanent medical implants. One possible solution is a zwitterionic polymer based on polysaccharides, which is a practical approach to overcoming this disadvantage. It combines the advantages of zwitterionic polymers and polysaccharides to avoid thrombosis and resist non-specific protein adsorption (Yao et al., 2021). Several possible solutions are reported to overcome or avoid issues with classic zwitterion coatings. For that reason, poly (sulfobetaine methacrylate) (polySBMA) has emerged as a favorable choice among the new generation of zwitterionic polymers due to its low cost, simple synthetic procedures, and ability to produce a strong hydration surface on the coated material. However, this technique still needs to be optimized to be applied commercially in CMDs (Hedayati et al., 2019).

#### 4.1.3 Textured surfaces

Texturing is another approach to improve the hemocompatibility of blood-contacting materials. Surface morphology and mechanical characteristics influence material-cell interactions. The impact of various textures differs according to cell type and the extent, type, and scale of topographic patterns (Kyriakides, 2015). Textured surfaces promote the formation of a stable biological lining known as a pseudo-neointimal layer of adsorbed and entrap denatured proteins, platelets, fibrin, and red blood cells which accounts for hemocompatibility (see Figure 2E) (Zapanta et al., 2006; Jing et al., 2007). Also, it has been reported the size, depth, and distribution of cavities on the material surfaces may significantly impact neointimal adhesion and reduce thromboembolic challenges (Zapanta et al., 2006). Several techniques are used to modify surface topography such as photolithography, soft lithography, chemical vapor deposition, laser texturing, ion beam lithography, and electron beam lithography (Riveiro et al., 2018). These techniques are classified between processing rate, processing steps, chemical products employed, treated area, and costs (see Figure 2E).

Photolithography is the technique of transferring patterns from a photomask to a photoresist using light. In biomedical applications, photolithography uses the following methodology: 1) the wafer is washed to eliminate any foreign matter, 2) spin coating the photoresist (e.g., monomers, oligomers, or polymers) onto the wafer, 3) the photomask is positioned on top of the photoresist, later the UV light is allowed to pass through the mask and finally 4) solvents are used to remove the unexposed area, leaving the desired patterns (Tran and Nguyen, 2017). On the other hand, soft lithography (SL) uses the principles of lithography; the main difference between photolithography and SL involves the working material. SL is designed to work in organic materials and polymers. SL prints onto a surface or a mold and shapes a polymeric film using an elastomeric stamping process or photomasks to create or mimic structures (Qin et al., 2010; Tran and Nguyen, 2017). On the other hand, electron beam lithography uses electrons to print, while photolithography uses light. Electron beam lithography is used for exposure. The beam is accurately directed to positions on the wafer to form the pattern. The electron beam exposes the pattern dot by dot and line by line, which can be more time-consuming than photolithography. However, it is a more flexible system and does not require expensive masks (Qin et al., 2010). On the other hand, ion beam lithography uses ions instead of electrons, this technique has a higher resolution than electron beam lithography. Ion beam lithography can be employed in two forms, in direct writing format, and with a mask. The most popular method is to use a mask (Tran and Nguyen, 2017). Lastly, chemical vapor deposition employs the following core process: 1) the thin layer is deposited on a base material (substrate), such as glass or silicon wafers, according to vapor deposition methods; 2) the substrate is subjected to one or more vaporized sources, which, coupled with other stimuli (heat or plasma), create a thin coating on the substrate’s surface. The chemistry of the vapor determines the sort of thin film that will deposit (Liu et al., 2023).

Among all the texturing surface techniques described before, laser texturing represents the greatest option to perform or create textures on surface of materials such as polymers and metals. One advantage is the ability to modify surface roughness and chemistry in a single step without harmful agents; at the macro, micro, and nano levels, with high spatial and temporal precision (Tran and Nguyen, 2017; Wu et al., 2023). Furthermore, has a fast-processing rate and only one processing step; large areas can be treated and can be made with medium costs. Laser deposition is the technique most employed in texture surface (Zapanta et al., 2006; Riveiro et al., 2018; Dong et al., 2022; Malone et al., 2022; Rahvar et al., 2022; Schieber et al., 2022; Wang and Ren, 2022; Yi et al., 2022). In general, CMDs with textured blood-contacting surfaces have demonstrated good hemocompatibility. There is less thrombus formation on textured surfaces compared to non-textured surfaces (Fujisawa et al., 1999). For that reason, textured surfaces are commercially applied on VADs (HeartMate ^®^ II) (Griffith et al., 2001) (Zhao and Feng, 2020).

### 4.2 Bioactive surfaces

Bioactive surfaces can prevent serious problems (coagulation, platelet adhesion, thrombus formation, and inflammation) and not only avoid protein absorption like passive coatings. Bioactive surfaces are coatings that employ the attachment of bioactive compounds, including coagulation inhibitors, antiplatelet agents, fibrinolytic agents, and biopolymers (e.g., polysaccharides, peptides, enzymes, and antibodies) (Amiji and Park, 1993; Kidane et al., 2004; Rotmans et al., 2005; Rau et al., 2007; Murugesan et al., 2008).

#### 4.2.1 Albumin coatings

Albumin protein performs critical metabolic functions such as regulating oncotic pressure, binding, and transporting various molecules (van de Wouw and Joles, 2022). Albumin rapidly binds to hydrophobic surfaces and lacks peptide sequences that allow it to interact with platelets, leukocytes, and coagulation cascade enzymes (Amiji and Park, 1993; Lin and Hlady, 1994; Brynda et al., 2000; Labarrere et al., 2020).

There are three general methods for developing an albumin-coated surface (see Figure 2F). The first method is adsorption on hydrophobic surfaces. For instance, it has been reported the attachment of albumin under a polydopamine surface on a polyethylene membrane using a mussel-inspired coating. This technique showed an enhanced capability to resist protein fouling and improve blood compatibility (Fischer et al., 2018). However, it has been reported in biological environments that immobilized albumin is probably denatured and displaced by other proteins (Barberi and Spriano, 2021). The second method is binding the albumin to other compounds; this method was designed to avoid albumin denaturation. In this method, albumin is reversibly immobilized to the surface by incorporating sulfonic acid, antibodies, bilirubin, or octadecyldimethylsilyl (C18) alkyl chains (Lin and Hlady, 1994; Gonçalves et al., 2009; Fischer et al., 2012). For instance, it has been reported that poly (2-hydroxyethyl methacrylate) (pHEMA) hydrogel films are modified with aliphatic C18 chains, taking full advantage of albumin’s mechanism as a fatty acid carrier and affinity for alkyl chains. Albumin was reversibly adsorbed at physiological conformation by C18 alkyl chain grafted surfaces, designed to allow for low platelet activation with reduced complement activation, leukocyte adhesion, and coagulation stimulation (Gonçalves et al., 2009; Fischer et al., 2012). On the other hand, the third method consists of self-assembled albumin conjugates. In this method, self-assembled albumin is designed to avoid unfordable or weak chemistry between the polymer chain and the albumin, using an intermediate which is a linker, the linker will provide a strong attachment of the albumin, but this technique still needs to be optimized to be applied in CMDs commercially (see Table 2) (Gonçalves et al., 2011; Tao et al., 2019; Kuten Pella et al., 2022). Albumin coatings are mainly designed for polymeric materials of CMDs (e.g., polytetrafluoroethylene, polydimethylsiloxanes, polyurethane, polycarbonate), but at the moment, these coatings need to be improved to be applied in medical devices or CMDs (Yamada et al., 2014; Cheng et al., 2019). One approach to solve this inconvenience is the combination of multiple compounds (e.g., heparin, elastin, and fibrinogen) linked with albumin to produce a suitable matrix (Manabe and Nara, 2021).

#### 4.2.2 Anticoagulant surfaces

Heparin immobilization is one of the most chemical compounds utilized to enhance biocompatibility in CMDs. Heparin’s anticoagulant activity is caused by catalyzing the inhibitory activity of the protein antithrombin III (AT III), which inhibits thrombin and the coagulation factor (FXa), as well as other coagulation factors (Hedayati et al., 2019). Heparin is an indirect thrombin inhibitor that binds to AT III through a pentasaccharide sequence (see Figure 2G). (Hedayati et al., 2019; Badv et al., 2020). The interaction of the heparin and the formation of the complex Thrombin—antithrombin is the foundation of the Carmeda^®^ BioActive Surface technology that is commercially applied on polymers (e.g., polytetrafluoroethylene) (Begovac et al., 2003). Multiple immobilization strategies have been reported to immobilize heparin onto metal or polymer cardiovascular materials such as stain steel 316L, polytetrafluoroethylene, polyethylene terephthalate, polyurethanes, poly (lactic acid), polypyrrole, and polysulfone (Qi et al., 2013).

Heparin is an anionic polysaccharide containing functional groups that include carboxylic acid, sulfonic, and sulfanilamide functional groups (Badv et al., 2020). The functional groups of heparin allow the immobilization to supporting matrices via: 1) electrostatic absorption (based on the interaction between the negatively-charged sulfate groups of heparin with positively-charged groups) on the material surface, 2) covalent immobilization (through different surface conjugations of functional groups of heparin), and 3) recently integration into hydrogels or insertion into release systems (see Figure 2G) (Murugesan et al., 2008; Liu et al., 2014a; He et al., 2017). One disadvantage of heparin coatings is due to structural changes in heparin caused by immobilization reactions and cannot bond properly with AT III (Murugesan et al., 2008; Fischer et al., 2018; Badv et al., 2020). To solve heparin activity due to the immobilization technique, several strategies for preserving heparin’s biological activity have been applied. In the endpoint technique, heparin is immobilized via its aldehyde group to allow the coupling to a primary amino group on the surface of the material (Jaffer et al., 2015; Fischer et al., 2018). While in spacers technique (e.g., PEG or PEO), the heparin becomes more accessible for interactions with AT III and the heparin molecule’s steric hindrance is reduced (Fischer et al., 2018). Finally, in cross-linked multilayers technique, heparin is immobilized with other active molecules as: fibronectin, phosphorylcholine, chitosan, alginate, collagen, and dexamethasone (Fischer et al., 2018; Badv et al., 2020).

On the other hand, not only the conjugation process is a critical step, also heparin’s lifetime is limited due to its biodegradability *in vivo* (Fischer et al., 2018). Furthermore, heparin has a proclivity to bind other plasma proteins than AT III (e.g., platelet factor 4, histidine-rich glycoprotein, vitronectin, fibronectin, and lipoproteins, albumin), which may lead to a loss of anticoagulant activity (Fischer et al., 2018). Heparin has several negative side effects, including hemorrhagic complications and heparin-induced thrombocytopenia (see Table 2) (Fischer et al., 2018); led to the creation of alternative heparin analogs (e.g., hirudin, bivalirudin) (Qi et al., 2013), anticoagulant proteins (thrombomodulin, activated protein C, tissue factor pathway inhibitor) (Badv et al., 2020; Chan et al., 2004; Han et al., 2001; Kador et al., 2011; Lu et al., 2012; Lukovic et al., 2015) and contact system specific inhibitors (corn trypsin inhibitor, inhibitor of FXIIa) (Fischer et al., 2018; Badv et al., 2020).

#### 4.2.3 Platelet inhibitors

Surface coatings using platelet inhibitors aim to inhibit platelet enzymes (cyclo-oxygenase) or receptors (e.g., ADP and GPIIb/IIIa receptors) that promote the activation of platelet aggregation on the surface of the CMDs (Chen et al., 2020; Nurden et al., 1999; Thon and Italiano, 2010; Xiang et al., 2019). The complete mechanism of platelet aggregation in CMDs was previously discussed in Section 3 (see Figure 1). Antiplatelet coatings, used to target platelet adhesion, activation, and aggregation, are based on prostaglandin E1 (PGE1), dipyridamole, apyrase immobilization, systems eluting the GPIIb/IIIa inhibitor (abciximab), and nitric oxide (NO)-releasing coatings.

For instance, dipyridamole inhibits platelet activity by inhibiting cyclic phosphodiesterase. Dipyridamole has covalently attached to polyurethane materials (Liu et al., 2014b). Another compound reported is prostaglandins (PGE1). This prostaglandin can inhibit platelet activity by increasing cyclic adenosine monophosphate through the augmented activity of adenylate cyclase. It has been reported coatings in polymers (polytetrafluoroethylene and dacron) (Liu et al., 2014b). Also, the immobilization of enzymes; apyrase enzyme has the ability to degrade adenosine diphosphate, a well-known platelet aggregation agent released from damaged red cells and platelets (see Table 2). This enzyme was immobilized in polystyrene, showing decreased platelet adhesion and activation (Liu et al., 2014b). On the other hand, other antiplatelet agents such as aspirin, vascular endothelial growth factor (VEGF), and GPIIb/IIIa receptor inhibitors have been reported (Nurden et al., 1999; Warner et al., 2011). GPIIb/IIIa has received attention because of their mode of reaction, which leads to blocking the receptor on activated platelets which mediate the adhesion of platelets to adsorbed fibrinogen on silicone surfaces.

On the other hand, nitric oxide (NO) is a signaling molecule that healthy endothelial cells release into the bloodstream, allowing the prevention of platelet activation, platelet aggregation, and anti-inflammation process, and also has anti-bacterial activity (Mowery et al., 2000; Yang et al., 2015). There are two primary strategies for surface local nitric oxide production. One strategy relies on a direct release mechanism in which NO donors diazeniumdiolates are immobilized (Gappa-Fahlenkamp and Lewis, 2005; Annich et al., 2013; Qi et al., 2014; Schanuel et al., 2015).The second strategy is to use catalytic agents that synthesize nitric oxide from physiological sources (s-nitrosoglutathion, s-nitrosocysteine, and s-nitrosoalbumin) (Duan and Lewis, 2002; Li et al., 2021). However, NO release does not confer efficiency for long-lasting anti-thrombogenicity on the blood-contacting material (Liu et al., 2014b). Coatings with other biological functions may be necessary, such as those provided by active anticoagulants. Also, it has been reported that excessive NO synthesis from exogenous sources could be harmful, implying higher interactions with superoxide to form peroxynitrites (Yang et al., 2015).

#### 4.2.4 Fibrinolytic coatings

Using fibrinolytic agents in surface modification of blood-contacting material will lead to thrombus degradation. The thrombus degradation process *in vivo* is triggered by a tissue-type plasminogen activator (tPA), which converts plasminogen to plasmin (see Figure 1 **)**. This conversion will allow the clearing of the surface of the blood-contact device due to the degradation of fibrin clot (Maitz et al., 2019; Sarode and Roy, 2019; Obstals et al., 2021). The use of fibrinolytic agents to develop a surface coating on the blood-contacting material is based on two main mechanisms. The first mechanism is designed to capture tPA. This is because tPA has lysine binding sites that can be used to attach tPA in lysine-functionalized surfaces (e.g., sulfonate groups). This type of coating has been reported with polyurethane surfaces. The second mechanism uses lysinized silica surfaces that allow attaching tPA in the bloodstream (McClung et al., 2003; Tang et al., 2013; Liu et al., 2014b). This last mechanism needs to be optimized and more research must be applied (see Table 2). A suitable approach has been reported in the anti-thrombosis therapy, with the potential use of fibrinolytic enzymes including plasmin, streptokinase, urokinase, recombinant tissue plasminogen activator, tenecteplase, and reteplase are the fibrinolytic enzymes clinically approved (Altaf et al., 2021).

### 4.3 Endothelialization

Natural endothelium is widely regarded as the ideal surface for blood contact materials. This is because the endothelium is the most physiological and hemocompatible blood-contacting material. The surface endothelialization has the advantage of producing a hemocompatible surface that is permanently active-allowing the expression of bioactive substances such as heparan sulfate, NO, thrombomodulin, and t-PA. The endothelialization of CMDs has become an appealing strategy (Mel et al., 2008; Figueiredo et al., 2019; Liu et al., 2014; Ren et al., 2015; Zhang et al., 2014).

There are two general methods to develop an endothelial surface (see Figure 2H). The first method involves *in vitro* cell seeding, for subsequent seeding on the material of the blood-contacting device before their implantation. However, it has been reported that several disadvantages are ligated to the process. For instance, it is very time-consuming, has a high production cost, and is challenging due to cell viability. To avoid all these limitations, the second method involves *in vivo* self-endothelialization using specific biomolecules (e.g., specific antibodies, DNA or peptide aptamers) anchored to the surface of the blood-contact material, allowing the attachment of endothelial progenitor cells. This attachment will lead to the endothelization of the blood-contact material (Aoki et al., 2005; Rotmans et al., 2005; Avci-Adali et al., 2010; Lim et al., 2011; Andukuri et al., 2013; Duan et al., 2019). One commercial example of the second method is stent coating with anti-CD34 antibodies, manufactured by Genous™, OrbusNeich-Medical Technologies (OrbusNeich^®^, 2022). Despite the advantages of this method, it has been reported that the lack of selectivity allows the growth of different types of cells rather than endothelial cells (see Table 2).

### 4.4 Antimicrobial coatings

A serious health concern is the antibiotic-resistant microorganisms with more than 5.3 million of deaths every year, and rising in the future (Shang et al., 2022). The antibiotic-resistant microorganisms have the ability to suppress antibiotics activity through three mechanisms: 1) the bacteria start releasing specific enzymes that destroy the antibiotics (e.g., penicillinase), 2) the permeability of the microbial walls for antibiotic diffusion is lowered, and 3) modifications in metabolic processes in the microbial cell (Mostafavi et al., 2022). Furthermore, not only these three mechanisms are a global concern, but also the ability to transfer resistant genes to other bacteria in their environment, successfully suppressing the effect of antibiotics and making them resistant to treatments (Mostafavi et al., 2022). Despite all the disadvantages of using antibiotics, antibiotic coatings of medical devices continue to be proposed (e.g., silver sulfadiazine, gentamicin, vancomycin, minocycline, rifampicin, amikacin, and chlorhexidine) (Mostafavi et al., 2022). Another approach is the development of antimicrobial hydrogels; which are interesting for medical devices and wound healing applications, and represent a better option than using only antibiotic coatings of blood-contacting material. For instance, it has been reported chitosan hydrogels grafted with PEG, antimicrobial polycarbonate hydrogels, and aldehyde-modified PEG hydrogel-loaded colistin which are examples of antifouling and antimicrobial agent-release hydrogels showing good efficiency on contact blood materials (Zhu et al., 2019). Another concern is the infection of polymeric devices or polymeric parts of CMDs devices; for that reason, antimicrobial and antifouling strategies emerge to prevent microbial adhesion or eliminate adhering bacteria (Zander and Becker, 2018) using multiple forms of brush forming polymer techniques (e.g., physical adsorption, covalent attachment, impregnation, migrating additives, and bulk incorporation) (Zander and Becker, 2018). Another technique is TYRX, where CMDs like ICDs are wrapped with the TYRX bag. This bag or antibacterial envelope is designed to elute minocycline and rifampicin into surrounding pocket tissue, protecting the device from local infection for at least 7 days after implantation (Tarakji et al., 2019; Mittal et al., 2020; Chaudhry et al., 2022). All these new strategies to combat bacterial infections involve antibiotics. Antibiotics are not considered a suitable option because, with time, the microorganisms will find the pathway to avoid antibiotics and the ability to transfer resistant genes to other microorganisms (Uddin et al., 2021; Tan et al., 2022). For that reason, it is mandatory to develop new surface functionalization strategies to combat bacterial infections.

Recently has emerged the use of antimicrobial peptides (AMPs) as a promising alternative to antibiotics in the formulation of surface coatings. AMPs have antibacterial activity against various microorganisms, including Gram-positive and Gram-negative bacteria, fungi, and some viruses (Li et al., 2018). AMPs are generally short, with 12–100 amino acids. AMPs have multiple physicochemical characteristics or “driving forces” that allow them to interact with microorganisms. Among all their physicochemical characteristics, it is important to highlight: 1) the net positive charge (due to the amino acids arginine, histidine, and lysine), between 2 and 9, improve the interaction with anionic lipids, 2) hydrophobicity necessary for membrane penetration, and 3) flexibility conformation and membrane interaction (Jenssen et al., 2006; Dini et al., 2022). These physicochemical characteristics are necessary for AMPs to function as antimicrobial agents and interact with bacterial membranes. Despite all advantages of AMPs, there are no examples of AMP coatings for CMDs. For this reason, it is necessary to develop new research in this area of interest.

## 5 Considerations

**FIGURE 3:**
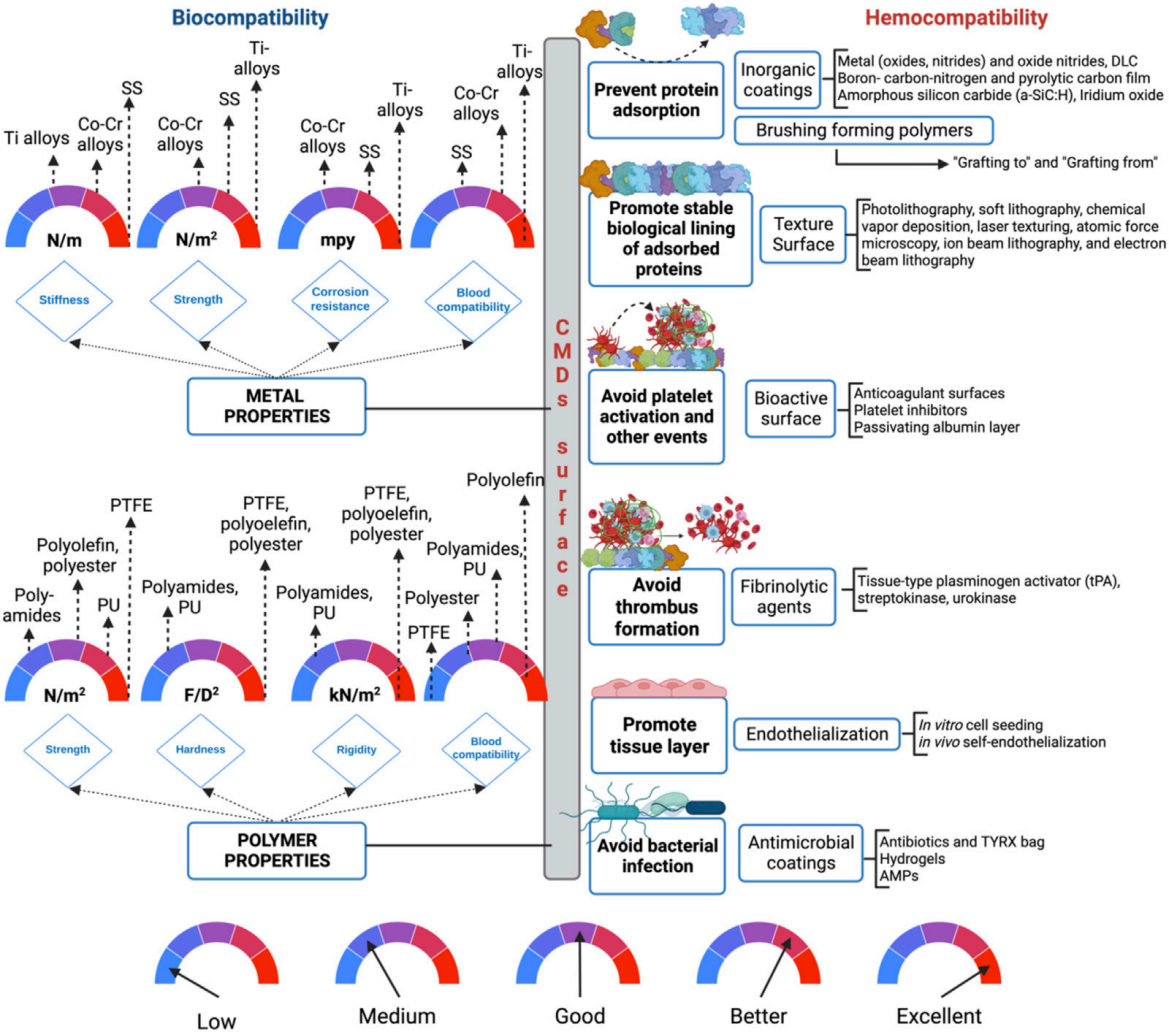
Considerations for the development of CMDs and the surface coatings. Abbreviations. SS, Stainless steel; PTFE, Polytetrafluoroethylen; PU, Polyurethane; AMPs, Antimicrobial peptides; N/m, Newtons per meter; N/m^2^, Newtons per square meter; MPY, Mils penetration per year; F/D^2^, Force per square diameter; kN/m^2^, Kilonewton per square meter.

Hemocompatibility and biocompatibility of materials are very important factors in the success of CMDs. Many risk factors regarding the low biocompatibility of materials (see Table 1) can be overcome by choosing them correctly. For instance, metals and alloys (stainless steel (SS), cobalt chromium (CoCr) alloys, and titanium (Ti) alloys) are highly applied in CMDs due to their characteristics (e.g., stiffness, strength, and corrosion resistance). Among all these materials, it is important to highlight that titanium alloys (e.g., nitinol) represent the best option, for all the characteristics mentioned previously (see Figure 3). Furthermore, nitinol has been reported as the best option material for blood compatibility. Another exciting feature of nitinol is the shape memory effect offered, which is commonly used to manufacture self-expanding memory stents (Jaganathan et al., 2014). On the other hand, polymers (e.g., polyamides, polyolefin, polyester, polytetrafluoroethylene, and polyurethanes) are also commonly used as implant materials in CMDs (see Figure 3). Polymers offer multiple characteristics such as: strength, hardness, and rigidity. Among all the polymers currently used in CMDs, it has been reported that polyolefin represents the best option. Furthermore, polyolefin offers better blood compatibility than the other polymers currently used (Jaganathan et al., 2014). However, the choice of materials often sacrifices the best biocompatibility option due to another characteristic that influences the correct functioning of the device.

There is a serious need to produce biomaterials that imitate the attributes of native heart tissues to avoid rejection by the host. One suitable solution to avoid rejection by the host is making the material biocompatible, which can be successfully achieved with innovative surface functionalization procedures. Surface modifications can enhance hemocompatibility, avoiding the irreversible adsorption of proteins to the material’s surface. Specifically, inorganic coatings, brush-forming polymers, and zwitterion polymers have demonstrated to be a suitable option to avoid protein adsorption due to the generation of hydrophilic surfaces, repellent surface charges, hydration forces, and steric hindrance (see Figure 3; Figures 3A,B). On the other hand, when the protein adsorption is the goal to form a pseudo-neointimal layer that accounts for hemocompatibility, surface texture is a suitable option (see Figure 2E), because the pseudo-neointimal layer avoids the irreversible protein adsorption that activates platelets and further reactions. Another surface modification used to avoid protein adsorption and platelet activation involves the generation of a tissue layer (endothelization) on the surface of the CMDs (see Figure 2H). Surface modifications that focus specifically on avoiding platelet activation, are called bioactive surfaces (see Figures 2F,G), because this type of surface modification employs the use of anticoagulants and platelet inhibitors. Moreover, if the desired goal is to avoid thrombus formation, there are still alternatives to initiate fibrinolysis (see Figure 1; Figure 3). Finally, it is worth mentioning that microbial infection is becoming a serious concern in global health, especially when it comes to CMDs. Currently, antimicrobial coatings used for CMDs are based on antibiotics and AMPs (see Figure 3) some of them are commercially available. Additionally, AMPs can be a great alternative to avoid the antibiotic resistance. In summary, surface modifications of CMDs are currently a great challenge that needs to take into account the advantages and disadvantages described before (see Table 2), which need to be carefully considered in order to apply them to the different surface materials.

## 6 Conclusion and future perspectives

The design of blood-compatible materials requires understanding the physiological mechanisms that give rise to undesirable blood-material interactions. In particular, the materials used in the design of CMDs are determined by the disease for which the CMDs are intended. For example, we review the utility of polymers in developing CMDs when the necessary materials need to be flexible and durable. On the other hand, when there is a need of materials with high mechanical resistance or more remarkable conductors, metals, and metals alloys are the best option. Also, the combination of these two types of materials is used to develop and enhance the ability to perform high workflow rates in CMDs. Even though metals and polymers have tremendous advantages in developing CMDs, there is a predominant recurring challenge, which is biocompatibility. For instance, metal alloys like nitinol, represent the best available material to choose if the desired characteristic is to enhance stiffness, strength, corrosion resistance, and blood compatibility (Jaganathan et al., 2014). On the other hand, polymers like polyolefin also represent an excellent suitable option, if the desired characteristic is strength, hardness, rigidity, and blood compatibility. As we review here, the combination of multiple materials is very good for the performance of the CMDs but makes more challenging its biocompatibility for short or long-term application. Currently, to enhance biocompatibility different surface functionalization strategies are being developed with promising performances. As described here, there are different surface coatings being explored, some of them already used in the market, and can be classified in different categories depending on which problem is overcoming; such as protein absorption, platelet activation, coagulation, or antimicrobial infections.

To achieve better biocompatibility and better-desired characteristics, the correct material selection is the golden key. We noticed that the combination of metals like nitinol and polymers like polyolefin could result in excellent CMDs performance without sacrificing biocompatibility. On the other hand, the combination of multiple surface modifications with different activities represents an area of interest. For instance, one suitable combination of surface modifications could be the use of antimicrobial surfaces such as AMPs and surface anticoagulants. This combination could enhance biocompatibility and avoid infections after implantation at the same time. However, most AMPs lack therapeutic usefulness due to physiological sensitivities or toxicity toward mammalian cells or the kidneys (Shang et al., 2022). For that reason, efforts to find safe and efficient procedures are required to move toward its real application. The current goal to achieve this is to develop a suitable matrix that can support both components. One possible approach could be the development of a matrix made of polymers such as PEG covalently anchored on the different materials of the devices. Several strategies are being developed to link bioactive proteins to PEG via click reactions or PEGylation (Turecek et al., 2016). To successfully achieve these strategies, considerations like pH conjugation, temperature, molar ratio, and time must be considered. On the other hand, it has been reported that PEGylation drops the activity but keeps the activity constant for a longer period (Ko and Maynard, 2018) which is an interesting characteristic to be taken into account for its application. More complex strategies could involve the use of thermoresponsive matrices that expose the bioactive molecules only upon activation (Zhu et al., 2019). This type of matrix could switch from hydrophilic lubrication in the CMDs to infection defense and anticoagulant activity. However, more efforts must be made to develop such complex coatings, taking into account key parameters like temperature, molar ratios, pH, and response time.

## Figures and Tables

**Table:** 

**AF**	Atrial fibrillation
**AMPs**	Antimicrobial peptides
**ASD**	Atrial septal defects
**AT III**	antithrombin III
**C18**	Octadecyldimethylsilyl
**CE**	Conformité Européenne
**CHF**	Congestive heart failure
**CMDs**	Cardiac medical devices
**CVDs**	Cardiovascular diseases
**DES**	Drug eluting stents
**DLC**	Diamond like carbon
**FDA**	Food drug administration
**HF**	Heart failure
**ICDs**	implantable cardioverter-defibrillators
**LAA**	Left atrial appendage
**LAAO**	left atrial appendage occlusion
**MPC**	2-methacryloyloxyethyl phosphorylcholine
**NMP**	Nitroxide-mediated radical polymerization
**pDA**	Poly(dopamine)
**PEG**	Polyethylene glycol
**PGE1**	prostaglandin E1
**pHEMA**	Poly(2-hydroxyethyl methacrylate)
**PM**	Pacemakers
**polySBMA**	Poly(sulfobetaine methacrylate)
**PyC**	Pyrolytic carbon
**RAFT**	Reversible addition-fragmentation polymerization
**ROMP**	Ring-opening metathesis polymerization
**SL**	Soft lithography
**stMCS**	Short-term mechanical circulatory supports
**TAH**	Total artificial hearts
**TAVI**	Transcatheter aortic valve implantation
**tPA**	Tissue-type plasminogen activator
**VADs**	Ventricular assist devices
